# Composite Nanofibers Containing Multiwall Carbon Nanotubes as Biodegradable Membranes in Reconstructive Medicine

**DOI:** 10.3390/nano9010063

**Published:** 2019-01-04

**Authors:** Andrzej Hudecki, Dorota Łyko-Morawska, Wirginia Likus, Magdalena Skonieczna, Jarosław Markowski, Renata Wilk, Aleksandra Kolano-Burian, Wojciech Maziarz, Jolanta Adamska, Marek J. Łos

**Affiliations:** 1 Institute of Non-Ferrous Metals, 44-100 Gliwice, Poland; aleksandra.kolano@imn.gliwice.pl; 2 Katedra Chirurgii Czaszkowo-Szczękowo-Twarzowej i Chirurgii Stomatologicznej, Śląski Uniwersytet Medyczny w Katowicach, 40-027 Katowice, Poland; dorota.lyko@gmail.com; 3 Department of Anatomy, School of Health Sciences in Katowice, Medical University of Silesia, 40-055 Katowice, Poland; wirginia.likus@gmail.com (W.L.); renatawilk@poczta.onet.pl (R.W.); 4 Biosystems Group, Institute of Automatic Control, Faculty of Automatics, Electronics and Informatics, and Biotechnology Centre, Silesian University of Technology, 44-100 Gliwice, Poland; Magdalena.Skonieczna@polsl.pl; 5 ENT Department, School of Medicine in Katowice, Medical University of Silesia in Katowice, 40-055 Katowice, Poland; jmarkow1@poczta.onet.pl; 6 Institute of Metallurgy and Material Science Polish Academy of Sciences, 30-059 Kraków, Poland; wojto17@hotmail.com; 7 Wydział Farmaceutyczny z Oddziałem Medycyny Laboratoryjnej Śląski, Zakład Biologii Molekularnej Katedry Biologii Molekularnej, Uniwersytet Medyczny w Katowicach, 40-055 Katowice, Poland; jolaa@sum.edu.pl; 8 Centre of Biotechnology, Silesian University of Technology, 44-100 Gliwice, Poland; 9 Centre de Biophysique Moléculaire, UPR4301 CNRS CS80054, Rue Charles Sadron, 45071 Orleans CEDEX 2, France

**Keywords:** electrospinning, multiwall carbon nanotubes, polycaprolactone, reconstructive medicine, titanium

## Abstract

We have tested titanium (Ti) plates that are used for bone reconstruction in maxillofacial surgery, in combination with five types of novel long-resorbable biomaterials: (i) PCL_0_—polycaprolactone without additives, (ii) PCL_MWCNT_—polycaprolactone with the addition of multiwall carbon nanotubes (MWCNT), (iii) PCL_OH_—polycaprolactone doped with multiwall carbon nanotubes (MWCNT) containing –OH hydroxyl groups, (iv) PCL_COOH_—polycaprolactone with the addition of multiwall carbon nanotubes (MWCNT) containing carboxyl groups, and (v) PCL_TI_—polycaprolactone with the addition of Ti nanoparticles. The structure and properties of the obtained materials have been examined with the use of Scanning Electron Microscopy (SEM), Transmission Electron Microscopy (TEM), Raman spectroscopy, Fourier transform infrared spectroscopy (FTIR), and/or X-ray powder diffraction (XRD). Titanium BR plates have been covered with: (i) PCL_0_ fibers (PCL_0BR_—connection plates), (ii) PCL_MWCNT_ fibers (PCL_MWCNTBR_—plates), (iii) PCL_OH_ fibers (PCL_OHBR_—plates), (iv) PCL_COOH_ (PCL_COOHBR_—plates), (v) PCL_TI_ fiber (PCL_TIBR_—connection plates). Such modified titanium plates were exposed to X-ray doses corresponding to those applied in head and neck tumor treatment. The potential leaching of toxic materials upon the irradiation of such modified titanium plates, and their effect on normal human dermal fibroblasts (NHDF) have been assessed by MTT assay. The presented results show variable biological responses depending on the modifications to titanium plates.

## 1. Introduction

The surgical reconstruction of bony defects in the mandible is a routine procedure performed for the rehabilitation of patients who require the reconstruction of the continuity of the mandible, due to impairment from injury or removal of the tumor [1]. The mandible plays an important role in chewing and swallowing; it is also important for vocalization [2,3,4]. It is a part of the stomatognathic system [2], and also provides the contour of the lower face, significantly affecting the aesthetics of the face [5].

The restoration of all of the mandibular recesses resulting from surgery is the primary goal of reconstructive surgery. Surgical techniques have been improving over the years through the use of various types of bone and soft tissue transplantations [6,7,8] fixed to titanium reconstruction plates, or the currently popular individualized bone prostheses made using CAD/CAM (computer-aided design/computer-aided manufacturing) techniques [9]. In clinical practice, the choice of the appropriate titanium plate, selection of the right size of the plate depending on the technique used, and the method of adjusting the shape of the plate so that the lower part of the craniofacial region is reproduced appropriately, are crucial for the success of the therapeutic process and for reducing the risk of subsequent complications. The use of titanium reconstructive plates in surgical practice may be accompanied by potential complications such as breaking the plate, losing the connecting screws [10], or—the worst scenario from the patient’s point of view—the exposure of reconstructive plates and development of fistulas connecting the oral cavity with the external environment [11,12,13]. These complications are much more common in large bone defects, especially when the resection of the mandibular body in the anterior segment is necessary. This process occurs in the early stages after surgery, with post-surgery tension in the tissues in the area of surgery and the lack of overgrowth of soft tissues (muscles, subcutaneous tissue) on the surface of the titanium plate. Furthermore, radiation therapy has a significant influence on the exposure of reconstructive plates later on [14]. Disfigured wounds with visible titanium fragments from the reconstruction negatively influence the patient’s well-being and psyche, causing increased post-operative depression; they also lower the public acceptance of such patients, which leads to the isolation of this group of patients and withdrawal from social life [15]. The continuation of treatment after tissue resection consists of filling the resulting cavity with a synthetic material and then irradiating the place where the tissue has been removed. Therefore, radiotherapy is meant to protect the place from which the tumor was excised before the tumor recurs, and eliminate any micrometastases that may have resided in the remaining tissue [16,17,18].

Modern regenerative medicine tries to find methods and materials that will make it possible to reconstruct and restore functions of damaged tissue, and will be able to protect the tissue environment over the time of tissue regeneration. These tasks are executed, among others, with the use of biodegradable polymeric materials of synthetic origin such as PLA [19], PLGA [15], PCL [20,21], or natural ones, such as chitosan, collagen, or hyaluronic acid [22]. The replacement of non-biodegradable implants with implants made of biodegradable materials that undergo total resorption reduces the likelihood of a reoperation. After implantation into the body of the recipient, the polymers are degraded to products that are easily removed by the human body, among others, through the citric acid cycle, which is also called the Krebs cycle [23]. The use of synthetic polymers is convenient due to their availability and chemical purity, while the popularity of natural polymers results from their biocompatibility. In practice, the most common combinations are: (i) two different synthetic polymers of different hydrophilic, electroconductive properties, and bio-stability such as PVA (polyvinyl alcohol) and PANI (polianiline) [24], PVP (Poli(winylopirolidon), and PEO/PEG (polyethylene oxide/polyethylene glycol) [25]; (ii) two natural polymers with differing properties such as chitin and chitosan [26]; (iii) synthetic polymers with natural polymers such as PCL (polycaprolactone) with chitosan [27,28]; or (iv) adding organic or inorganic additives to natural/synthetic polymer. The use of biodegradable polymeric materials should recreate the natural tissue scaffold—ECM (extracellular matrix), which includes both organic and inorganic components combined in a synergistic way with the cells of a given tissue [29]. A scaffold is an integral part of every tissue, as it supports the cells, gives tissues its elasticity and strength, and allows for the adhesion of cells, as well as their proliferation, and migration. The majority of works currently executed concentrate on designing and obtaining scaffolds and using various production technologies such as 3D printing, electrospinning, SLS, SLM, (selective laser sintering, selective laser melting) and natural as well as synthetic materials. Various doping materials have been introduced to the “basic” building blocks of the nanofibers mentioned above; i.e., multiwall carbon nanotubes (MWCNT) and graphene nanoplatelets (GNP) [30,31]. Several sources of cells are now available for tissue reconstitution (seeding of cells on scaffolds). These include cells obtained by reprogramming and by transdifferentiation [32,33,34], which are sourced from various tissues.

Using clinical data, our interdisciplinary scientific team tried to answer the question of “whether coating titanium plates with membranes obtained from micro- and nanofibers could be used as a solution to promote the, adherence and growth of cells on the implant surface, and hence, preventing the titanium plates from being exposed after surgery”.

## 2. Experimental Procedures

### 2.1. Materials Used to Obtain Composite Nanofibers

Biodegradable PCL polycaprolactone MW = 96,000 by Sigma Aldrich (Poznan, Poland) was used to obtain composite fibers. The solvent used was a 99.9% formic acid mixed with 99.9% pure acetic acid from Sigma Aldrich. The following were used as additives for nanofibers: (i) multiwall nanocomposite MWCNT-nanotubes from SSNANO, (Houston, TX, USA) 50–100 nm in diameter; (ii) SSNANO multiwall nanotubes from SSNANO, 50–100 nm in diameter, functionalized by –OH groups; (iii) MWCNT multiwall nanotubes by SSNANO with a diameter of 50–100 nm, functionalized with carboxylic groups –COOH, as well as (iv) titanium (Ti) nanomolecules with a diameter of 20–30 nm by SSNANO, to compare the biological response. Initially, MWCNT nanotubes of various diameters (<8 nm, 20–30 nm, 50–100 nm) were tested for uniform distribution in nanofibers. MWCNTs that were 50–100 nm in diameter achieved the most homogenous distribution within the nanofibers; hence, such ones were chosen for further experiments.

### 2.2. Cell Culture Materials Used

In survival studies, NHDF cells—normal human dermal fibroblasts (LONZA)—were used. Cell cultures were carried out in DMEM-F12 medium (SIGMA), supplemented with 10% fetal bovine serum (FBS, SIGMA), under sterile and standard environmental conditions (37 °C, 60% humidity, 5% CO_2_).

### 2.3. Titanium Plates Used 

The titanium reconstruction plates used in the study were supplied by the Synthes z o. o. (Warsaw, Poland). They were 2.4 mm thick, and have been used routinely in reconstructive surgery of the head and neck, which require bending to fit the resulting mandibular loss during surgery and fixing with titanium screws to the bone. Surgical plates used in surgical practice are not covered with any other additional materials that may support their overgrowing with soft tissues.

### 2.4. Preparation of Solutions

In order to obtain composite nanofibers containing the mentioned additives, a mixture of solvents—formic acid and acetic acid in the ratio of 70:30 m/m—was prepared. For each mixture, the following have been introduced: (i) MWCNT carbon nanotubes with the diameter of 50–100 nm with the share of 1% (PCL_MWCNT_); (ii) MWCNT carbon nanotubes with the diameter of 50–100 nm, functionalized with –OH groups with the share of 1% (PCL_OH_); (iii) MWCNT nanotubes with the diameter of 50–100 nm, functionalized with –COOH groups with a share of 1% (PCL_COOH_); or (iv) titanium nanoparticles with the diameter of 20–30 nm with the share of 1% (PCL_TI_). The solutions were ultrasonically dispersed for 15 minutes, and then supplemented with polymer (PCL) and—with the use of a magnetic stirrer—the mixture was converted to a solution having the total concentration of: 63% formic acid, 27% acetic acid, and 10% additives (10% = nine PCL + 1% MWCNT_[pure or functionalized]_).

### 2.5. Preparation of Nanofibers

The resulting mixtures (m/m) of solvents (63% + 27%, please see previous paragraph), polymers (9%), and additives (1%) were placed in a solution tank where, with the use of an infusion pump, they were pressed into the working chamber of the electrospinning device and subjected to strong stretching in the electrostatic field for conversion into a fiber. The flow of the polymer solution stream was defined at the level of one mL/h. The stream of the obtained solutions was subjected to an electrostatic field with the voltage of one kV/cm. The electrospinning process was carried out in 35% humidity of the gas, at the temperature of 25 °C.

### 2.6. Coating of Titanium Prostheses with Composite Nanofibres (Membranes)

In order to obtain layered materials, PCL_0 BR_, PCL_OH BR_, PCL_COOH BR_, PCL_MWCNT BR_, PCL_TI BR_, and BR titanium plates were cleaned with acetone for five minutes, then dried and subsequently placed in the working chamber of the electrospinning device where the fibers were applied directly to the titanium plates under an electrostatic field.

### 2.7. X-Ray Exposure

In practice, depending on the area of irradiation exposure, the total doses of irradiation are different. For example, for the central nervous system, they amount to 70–80 Gy, for the mandible they add up to 70 Gy, and for the thyroid they add up to 45–50 Gy [35]. The dose is chosen so that it has a certain energy at the appropriate depth within the targeted tissue. For deep tumors, photon radiation is used to determine the so-called percentage depth dose. The distance from the skin to the irradiation site is defined as the area of increasing dose. Total doses are determined for individual parts of the body and tissues. In contrast, the normal doses are fractionated, i.e., divided into smaller ones to achieve the total dose after a few sessions.

The membranes obtained from PCL_0_, PCL_OH_, PCL_COOH_, PCL_MWCNT_, PCL_TI_ nanofibers, and titanium plates coated with PCL_0 BR_, PCL_OH_BR_, PCL_COOH_BR_, PCLM_WCNT_BR_, and PCL_TI_BR_ membranes were placed in separate tanks and immersed in phosphate buffer saline (PBS) solution. Each sample was then subjected to X-ray exposure at 72 Gray (using the Clinac 600 therapeutic accelerator, Oncology Institute, Gliwice, Poland), that is the maximum dose of X-ray used in surgical practice in the field of mandibular surgery.

The resulting liquid obtained after separation of PCL_0_, PCL_OH_, PCL_COOH_ PCL_MWCNT_, PCL_TI_, PCL_0_BR_, PCL_OH_BR_, PCL_COOH_BR,_ PCL_MWCNT_BR_, and PCL_TI_BR_ from the PBS (phosphate buffer saline, a type of a quasiphysiological saline) solution was tested using 2,5-diphenyl-2*H* tetrazolium bromide (MTT) (cell viability assay) tests to determine survival, as well as tests using ICP-AES (Inductively coupled plasma atomic emission spectroscopy) and IR spectroscopy.

The study of the irradiated solution was carried out to determine whether the composite material under the effect of the total doses of X-rays received by patients after the removal of squamous cell carcinoma in the region of the mandible of the head and neck, may affect the surrounding liquid, changing its properties, and thus affect the cells.

### 2.8. FTIR

Infrared spectroscopy (IR) was used to determine the presence of PCL functional groups. The presence of functional groups was determined from the spectra recorded using Nicolet IR spectrometer by Thermo Company. The samples were ground with KBr powder in the proportion of 0.1 mg of fiber to three g KBr; then, they were pressed into pellets, which were then placed in a desiccator with a moisture binder (silica gel) for 30 minutes at 40 °C. The samples were then subjected to scanning 128 times with the resolution of 4 cm^−1^, in the wave number ranging from 400 cm^−1^ to 4000 cm^−1^. 

### 2.9. Raman Spectroscopy

IR spectroscopy was used to confirm the presence of carbon nanotubes in the tested solutions. For this purpose, the obtained solution—after separation from the irradiated composite material—was combined with aqueous solution of polyvinyl alcohol (PVA), and then frozen at −20 °C for 24 h. After freezing, the obtained samples were placed in a vacuum oven to remove water, after which the resulting dried material was tested using Raman spectroscopy.

### 2.10. XRD

The X-ray diffraction measurements of selected samples were performed at ambient temperature using Rigaku MiniFlex 600 diffractometer (Rigaku Corporation, Tokyo, Japan) with Cu Kα radiation (λ = 1.5406 Å), a tube voltage of 40 kV, and a current of 15 mA using the D/teX Ultra silicon strip detector.

### 2.11. SEM

The topography of the obtained nanofibers was analyzed with the use of a Zeiss Supra scanning electron microscope, at different settings of acceleration voltage and magnification ranges, selected for optimal observation of samples.

### 2.12. TEM

To determine the structure of PCL_0_, PCL_OH_, PCL_COOH,_ PCL_MWCNT_, and PCL_TI_ nanofibers, samples of fibers were applied directly to the surface of the copper mesh and structure analysis was performed, using high resolution transmission electron microscope TEM at accelerating voltage up to 300 kV and the following modes: Fourier transform (FFT), inverse Fourier transformation (IFFT), using bright field detector (BF), high-resolution wide field detector of dark field (HAADF), and transmission scanning mode STEM. Transmission microscopy has also been used to assess the structure of carbon nanotubes and titanium nanoparticles.

### 2.13. BET and Langmuir

The assessment of BET and Langmuir specific surface area was executed using the gas adsorption method, and a nitrogen of purity of 5.0 was used as the measuring gas. The tests applied the specific surface area analyzer, i.e., Gemini VII 2390t, by the Micrometrics (Norcross, GA, U.S.A).

### 2.14. MTT 

The effects of titanium plates and biomaterial membranes on cell survival and proliferation were assessed by 3-(4,5-dimethyl-2-thiazolyl) 2,5-diphenyl-2*H* tetrazolium bromide (MTT; Sigma Aldrich) assay. The tested biomaterials were irradiated in PBS solution at summarized dose of 72 Gy. The biomaterials were soaked in PBS for 24 h (during and after irradiation). Such PBS solutions were used for MTT assay upon 1:1 dilution in fresh complete DMEM-F12 medium containing 10% FBS (referred further to as “1:1-diluted PBS”). The 1:1-diluted PBS that was previously in contact with the tested biomaterials was then used for toxicity assessment using NHDF as test cells. NHDF were seeded at 1 × 10^4^ cells/well in a flat-bottom 96-wells plate in fresh DMEM-F12 medium, supplemented with 10% FBS, 24 h prior the assay. The 1:1-diluted PBS samples were then incubated for next 72 h without further dilutions with NHDF to assess any possible toxicity (the inhibition of proliferation). Additional samples were incubated in full-supplemented DMEM-F12 medium (“control-medium”) or in PBS (“PBS-control”). Next, the 1:1-diluted PBS was removed from, and the cells were washed with PBS solution. Then, each well was filled with 20 µl (five mg/mL) of MTT solution, and incubated in the cell culture incubator for three hours. Next, the plates were centrifuged and supernatant was discarded. Formazon crystals were dissolved in izopropanol-HCl solution (1:1 ratio). The readings were performed at 570 nm and 630 nm using a spectrophotometer (Epoch, TKBiotek). Each sample was evaluated three to six times, all of the measurements were presented as mean ± SD, and finally, the absorbance values were expressed as a percentage change in the viability of the tested cells, relative to the “control-medium” cells [%]. The significance of any changes, according to the control and untreated cells, was calculated with *s*-Student’s test with *p* value < 0.001. The statistically significant changes presented in the figures were indicated by an asterisk (∗). The results were analyzed using MS Office version 2.5.0 and MS Excel 2007.

## 3. Results

The research concerning the obtained composite materials was carried out, using (XRD) X-ray diffraction, Raman spectroscopy, TEM transmission microscopy, SEM scanning electron microscopy, ICP-AES Atomic Emission Spectroscopy, FTIR infrared spectroscopy by BET and Langmuir methods using gas adsorption, as well as in vitro tests and MTT tests.

**Figure 1:**
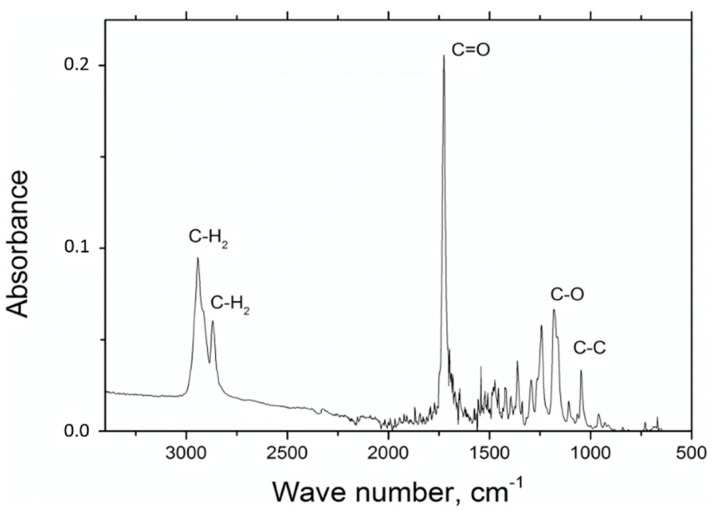
Fourier transform infrared (FTIR) spectrum for nanofibers polycaprolactone without additives.

In the experiments involving FTIR polycaprolactone spectroscopy, the presence of the following measures has been observed: C=O stretching vibrations for wave number s1720 cm^−1^, symmetric C–H_2_ stretching vibrations for wave number 2866 cm^−1^, asymmetric C–H_2_ stretching vibrations C–H_2_ for wave number 2943 cm^−1^, C–O and C–C for wave number 1294 cm^−1^ and C–O and C–C stretching vibrations for the wave number 1163 cm^−1^, which is characteristic for polycaprolactone (Figure 1).

**Figure 2:**
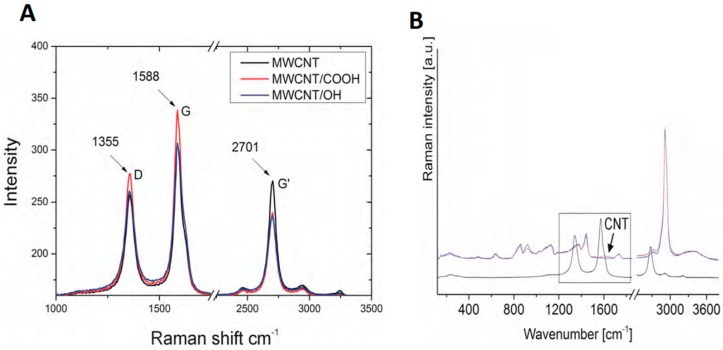
Raman spectrum for multiwall carbon nanotubes. (**A**) Raman spectrum for: MWCNT—multiwall carbon nanotubes 50–100 nm in diameter, MWCNT/COOH—multiwall carbon nanotubes 50–100 nm in diameter containing—carboxyl groups—COOH, MWCNT/OH—multiwall carbon nanotubes 50–100 nm in diameter containing OH hydroxyl groups. (**B**) The presence of carbon nanotubes was confirmed, due to the occurrence of the G-band for the frequency of ωG = 1588 cm^−1^ and the intensity of 306 for MWCNT. In the tested samples in first step, solution was mixed with PVA (polyvinyl alcohol), in the second step, the solution was frozen, and after that, water was removed by desiccation in vacuum.

Raman spectroscopy was used to examine the carbon nanotubes that were used in the experiments (Figure 2A,B). The tests revealed the presence of the G-band for the frequency of ωG = 1588 cm^−1^ and intensity of 306 for MWCNT, 306 for MWCNT/_OH_, and 338 for MWCNT/_COOH_, which proves the presence of graphite structures that are characteristic for the graphene and carbon nanotubes in the tested samples. The presence of the D-band was also confirmed for the frequency of ωD = 1355 cm^−1^ and intensity of 255 for MWCNT, 260 for MWCNT/_OH_, and 277 for MWCNT/_COOH_, confirming the occurrence of defects in the structure of carbon nanotubes. The tests also confirmed the presence of the G’ band with the frequency of 2704 cm^−1^ and an intensity of 270 for MWCNT, 263 for MWNCNT/_OH_, and 239 for MWCNT/_COOH_.

**Figure 3:**
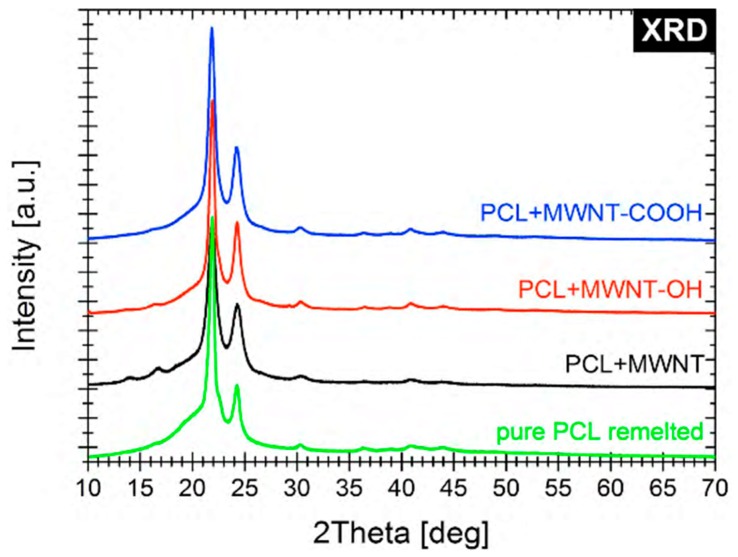
XRD spectra for: (I) pure polycaprolactone (PCL), (ii) PCLMWCNT—that is polycaprolactone with the addition of multiwall carbon nanotubes (MWCNT), (iii) PCLOH—that is polycaprolactone with the addition of multiwall carbon nanotubes (MWCNT) containing –OH hydroxyl groups, (iv) PCLCOOH—that is polycaprolactone with the addition of multiwall carbon nanotubes (MWCNT) containing carboxyl groups—COOH.

The XRD spectra confirmed the structures and functionalization of the biomaterials used in our experiments (Figure 3).

**Figure 4:**
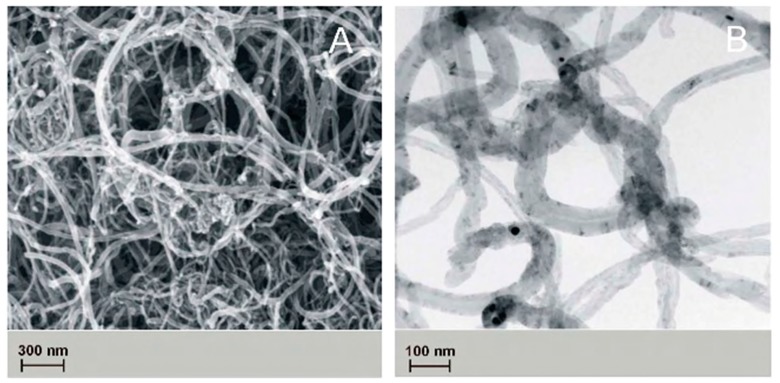
The ultrastructure multiwall carbon nanotubes: (**A**) images taken using a scanning electron microscope (SEM), (**B**) photographs taken using a transmission electron microscope (TEM).

The topography of the obtained nanofibers was analyzed with the use of scanning electron microscope, and the results are indicated in Figure 4.

**Figure 5:**
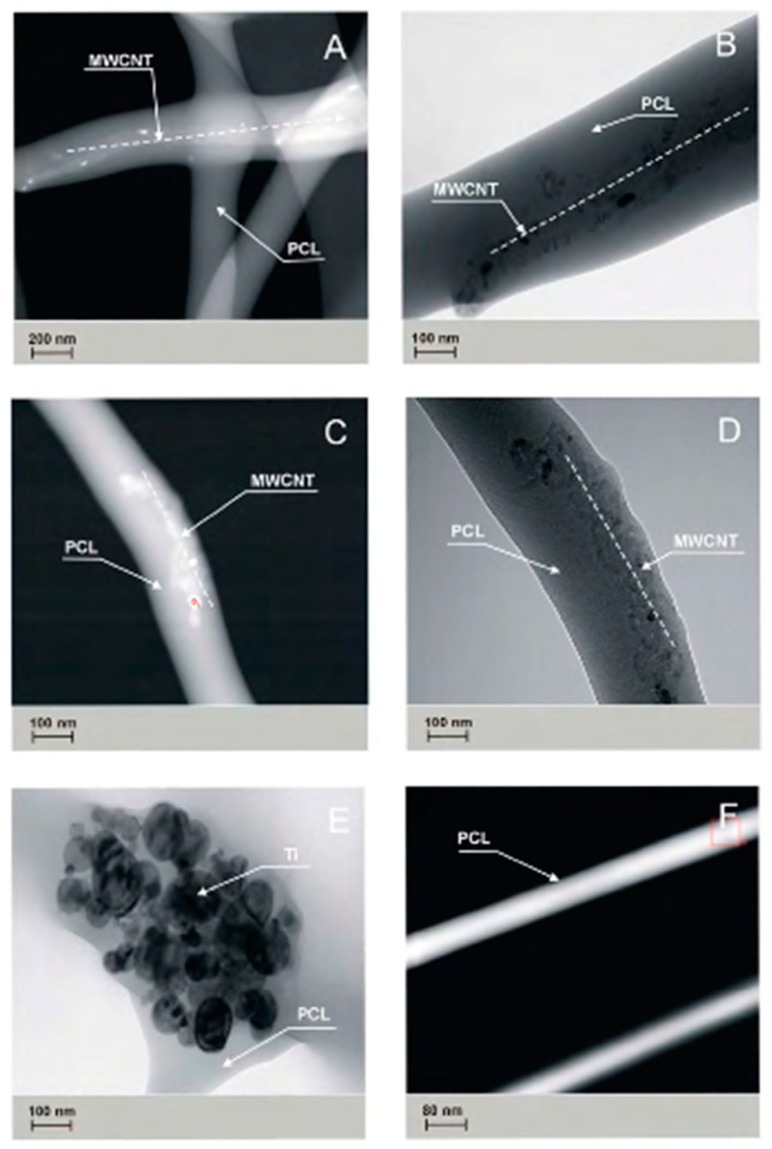
Ultrastructural features of generated nanofibers: (**A**) the structure of the PCL_MWCNT_ nanofibers observed in the TEM (BF mode), (**B**) the structure of the PCL_MWCNT_ nanofibers observed in the TEM (STEM-HAADF, mode), (**C**) the structure of the PCL_OH_ nanofibers observed in the TEM (BF mode), (**D**) the structure of the PCL_OH_ nanofibers observed in the TEM (STEM-HAADF mode), (**E**) the structure of the PCL_Ti_ nanofibers observed in the TEM (STEM-HAADF mode), (**F**) the structure of the PCL nanofibers observed in the TEM (STEM-HAADF mode). HAADF: high-resolution wide field detector of dark field.

The presence of carbon nanotubes and titanium nanoparticles has been confirmed in studies carried out on PCL_0_, PCL_OH_, PCL_COOH_, PCL_MWCNT_, and PCL_TI_ nanofibers, with the use of TEM transmission microscopy (Figure 5).

The PCL_OH_ and PCL_COOH_ samples showed a tendency of carbon nanotubes to accumulate closer to the surface of the elemental nanofiber, while in PCLM_WCNT_ samples, this trend was not observed. In the PCL_TI_ samples, the presence of Ti nanoparticle agglomerates in the tested material was confirmed. The studies performed using transmission TEM (Figure 5) and surface specific analysis by gas adsorption (Figure 5) shows a certain relationship between the observed structure of composite nanofibers and their surface properties. The study showed that by adding MWCNT carbon nanotubes to a solution, the specific surface area of fibers determined by BET and Langmuir increases up to 9.5 m^2^/g for BET and 17.25 m^2^/g for Langmuir, respectively. The observed increase in the specific surface is affected by a change in the electrical conductivity of the initial solution, which increases after the addition of carbon nanotubes. As a result, the solution with higher electrical conductivity undergoes a greater extension under the influence of the electrostatic field, which implies a reduction in the diameter of the obtained nanofibers and thus the observed increase in the BET surface area.

**Figure 6:**
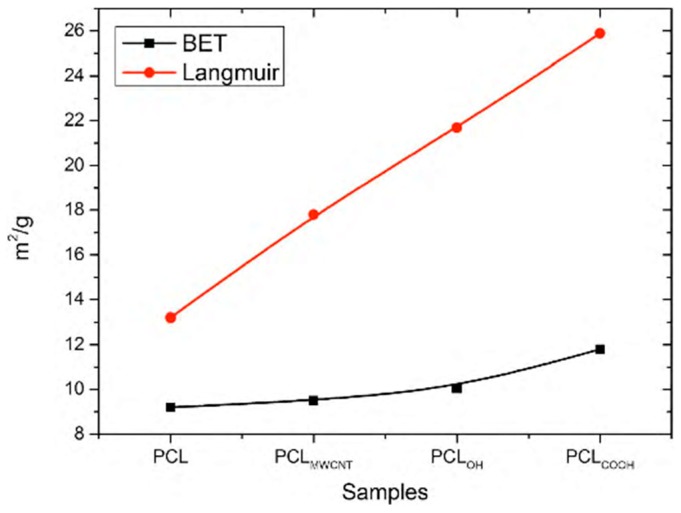
Surface area BET (m^2^/g) and Langmuir (m^2^/g) for: (i) pure PCL, (ii) PCL/MWCNT—that is polycaprolactone with the addition of multiwall carbon nanotubes (MWCNT), (iii) PCL/OH—that is polycaprolactone with the addition of multiwall carbon nanotubes (MWCNT) containing –OH hydroxyl groups, (iv) PCL/COOH—that is polycaprolactone with the addition of multiwall carbon nanotubes (MWCNT) containing carboxyl groups—COOH.

A much higher increase in the specific surface area is observed after the addition of MWCNT/_OH_ and MWCNT/_COOH_ functionalized nanotubes to the solution (Figure 6). In the tested samples, the specific surface area of fibers determined by the BET method increases, accordingly, to the level of 10.4 m^2^/g for PCL_OH_ and 11.89 m^2^/g for PCL_COOH_ and Langmuir 21.69 m^2^/g for PCL_OH_ and 25.85 m^2^/g for PCL_COOH_, respectively. Two properties are responsible for the increase in the surface area in both cases: (i) the first one, as in the case of PCL_MWCNT_ composite nanofibers, is the increase in the electrical conductivity of the solution contributing to the reduction of fiber diameter under the influence of the electrostatic field [36]; (ii) the second property that may promote the increase in the specific surface area of the fibers obtained using composite fibers is the interaction between the used mixture of carboxylic solvents (acetic acid and formic acid) and functional groups of carbon nanotubes, in this case hydroxyl groups –OH and carboxylic groups –COOH at the stage of conversion of the stream of solution into fibers. The carboxylic acids (formic and acetic one) used in the study help in the formation of hydrogen bonds between solvent groups and the functional groups of carbon nanotubes.

During the electrospinning process, the polymer solution stream is influenced by the electrostatic field; hence, an increase in conductivity entails stronger stretching of the polymer solution stream in the electrostatic field.

At the same time, in the electrospinning process, the particles of the solvent that is used evaporate from the surface of the stream. The formation of hydrogen bonds between the solvent particles and the functional groups of carbon nanotubes can promote their “drawing” toward the surface of the forming fiber, which leads to an increase in the specific surface area of the fibers, as observed in our experiments. This tendency was not observed in PCL_MWCNT_ samples, in which carbon nanotubes do not have functional groups (carboxyl and hydroxyl ones) on their surface, and were located in the entire volume of fibers.

In the case of the PCL_TI_ samples, a decrease in the specific surface area of the samples to 6.14 m^2^/g determined by the BET method, and to 9.29 m^2^/g for the specific surface area determined by the Langmuir method was observed (Figure 6). The observed decrease in the specific surface area of the fibers corresponds to the formation of agglomerates of Ti nanoparticles in the fiber structure, which consequently entails an increase in fiber diameter and a simultaneous decrease in the specific surface area.

Tests carried out using ICP-AES Atomic Absorption Spectroscopy confirmed the presence of carbon in the standard for PCL_0_ and PCL_Ti_ samples, but showed an increase in the presence of carbon to 299 mg/L for PCL_MWCNT_ samples, 156 mg/L for PCL_OH_ samples, and 146 mg/L. for PCL_COOH_ samples. Detected with the use of ICP-AES spectroscopy, the carbon came from the carbon nanotubes released into the solution, which was confirmed by Raman spectroscopy (Figure 2B). In the tested solution samples, the presence of carbon nanotubes was confirmed due to the occurrence of the G-band for the frequency of ωG = 1588 cm^−1^ and the intensity of 306 for MWCNT, which testifies to the occurrence of a graphite structure, which is characteristic for carbon nanotubes.

**Figure 7:**
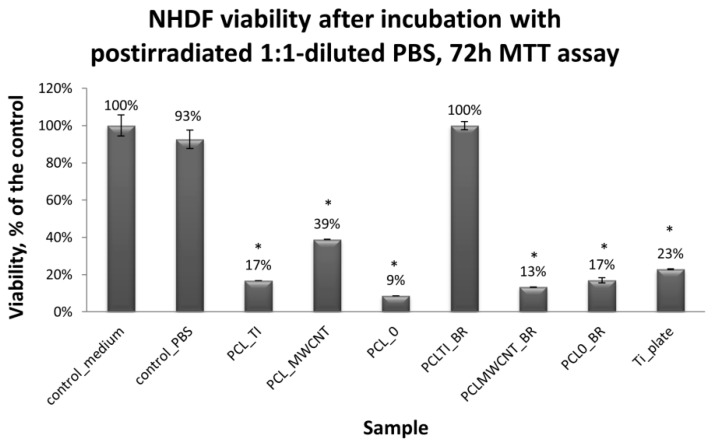
Normal human dermal fibroblasts (NHDF) cells viability after 72 h of 2,5-diphenyl-2*H* tetrazolium bromide (MTT) assay, results from three to six measurements presented as mean ± SD, and expressed as a percentage change in viability of tested cells, relative to the “control-medium” cells [%]. The significance according to the untreated cells was calculated with *s*-Student’s test with a *p* value < 0.001. Relevant changes are indicated by an asterisk (∗).

Cytotoxicity tests were carried out using MTT assay, in which biomaterial extracts (PBS solution) were tested. The NHDF viability in the presence of tested and irradiated membranes and plates covered with membranes were significantly reduced (Figure 7). However, in comparison to the titanium plates (Ti-plates), the obtained results, which are shown in Table 1, indicate some interesting effects. Hence, PBS extracts of samples exposed to X-ray (membranes or titanium plates coated with membranes) have a differential effect on the cells: (i) the survival of the cells in contact with PBS extracts in which the uncoated titanium plates were irradiated was considered the control; (ii) the survival of cells in contact with PBS extracts of PCL_0_ membranes decreased by 31.5% compared to the control sample; (iii) the survival of cells in contact with PBS extracts of PCL_0 BR_ samples decreased by 13%; and (iv) surprisingly, the survival of cells in contact with PBS extracts of PCL_MWCNT_ membranes increased by 34.4% compared to the control sample; (iv) but slightly decreased by 21% for PCL_MWCNT BR_, that is, for samples in which the titanium plates were covered with PCL_MWCNT_ membrane; (v) the survival of cells in contact with the PBS extracts of PCL_TI_ membranes decreased by 13.5% compared to the control sample, while it was significantly increased, by 268%, for PCL_TI_BR_ samples, that is, for samples in which the titanium plates were covered with PCL_TI_ membrane (Table 1).

## 4. Discussion

The study examined the structure and properties of tissue scaffolds (membranes) obtained by the combination of biodegradable polymer—polycaprolactone (PCL) containing carbon nanoparticles as an additive (multiwall carbon nanotubes, MWCNT) or metallic (titanium). Studies concerning the influence of X-rays used in radiotherapy on the structure and properties of membranes were also carried out.

Polycaprolactone was chosen due to the relatively long degradation time [20], which would be required in bone replacements due to the long time of bone tissue regeneration [37] while multiwalled carbon nanotubes were selected due to the possibility of the functionalization of their surfaces with bioactive groups, their usage as drug carriers [38], and possible offered improvements of mechanical properties.

Our experiments have shown that various combinations of biomaterials have different effects on the growth of human fibroblasts (NHDF).

Membranes containing the addition of PCL_MWCNT_ carbon nanotubes, PCL_OH_, or PCL_COOH_ do not show bioactive properties, such as a rapid stimulation of cell growth. Such property may be important for materials that are intended to replace tissue removed due to cancer, where materials not accelerating cell proliferation are desired, since increased proliferation would potentially increase the risk of the occurrence of secondary cancers.

On the other hand, carbon nanotubes are investigated by various scientific centers worldwide for their potential use in cancer therapy as carriers of bioactive agents [39]. Thus, combining carbon-containing nanotubes with biodegradable polymeric materials with diversified degradation time creates an opportunity to develop personalized therapies where the implant replacing surgically removed cancerous tissues, act as in situ reservoir of drug combination carefully selected to be most effective for that particular, individual cancer.

Among others, we tested how cells exposed to the liquid environment potentially containing substances released from tissue scaffolds are subjected to similar total X-ray doses that patients are treated with after the removal of bone tissue previously occupied by the tumor.

Similar types of composite nanofibers combined with therapeutic agents may be used in the future for medical therapies, among others, in treatments eliminating harmful microorganisms, creating favorable conditions for the development of desired tissues. Such effects may be achieved i.e., by developing the specific surface of nanofibers, allowing cell adhesion, as well as the proper porosity of nanofibers ensuring the passage of nutrients, oxygen, and metabolites, and—finally—by the controlled regeneration of bone and other tissues.

However, our study has some limitations, and the obtained results should be viewed in the context of the actual clinical procedures. For example, the fractionation of radiation used in radiotherapy—patients who take a total dose of 72 Gy—is typically split over 18 weeks, with the dose of four Gy per week. While in our study, the sample was subjected to a similar total radiation dose, it was acquired in a much shorter period. Moreover, depending on the area of the body, the doses may vary greatly, and may be i.e., lower than the tested in our experiments. The X-ray radiation used in cancer radiotherapy requires fractionation due to the biological effects exerted by ionizing radiation on tissues, by damaging the cell’s DNA and its other sensitive structures. Very high doses of radiation applied at one time can only lead to the necrosis of surrounding tissues. The radiosensitivity of the cells that are irradiated is of significant importance here. Cancer cells show radiation sensitivity depending on the type of tissue from which they originate, and carried mutations, but because of being rapidly dividing cells, they are generally more sensitive to radiation, compared to the surrounding healthy tissues. Proper selection of the dose that is necessary for effective treatment depends on the correct recognition of the cancer type. In the case of squamous cell carcinoma of the head and neck, its radiosensitivity is average; therefore, the total radiation dose is in the range from 45 Gy to 70 Gy, while in the case of the mandible, the tumor may be of a different origin. Therefore, usually the total dose is similar to the one used for tissues of low radiosensitivity, that is, above 70 Gy.

Radical radiation therapy assumes the complete recovery of patients; therefore, radiation fractions have to be chosen in a way to minimize the side effects for surrounding tissues. They are usually lower than those used for palliative radiotherapy, which is aimed at reducing the pain associated with the advancing cancer. The radiation used in the radiotherapy of the mandible is photovoltaic megavoltage radiation, usually with an intensity between 4–25 MeV.

One-time irradiation should not exceed several minutes. In the research carried out, the high single dose was only used to control the influence of all of the factors that can be applied to materials after they were implanted in the patient’s body. In the planned research, we will investigate to what extent radiotherapy can affect the overgrowth of cells within implants in various combinations of materials used depending on: (i) the irradiation site; in the current study, the samples were irradiated in laboratory dishes (in vitro tests); in practice, an implant is placed in a living organism (in vivo); (ii) the properties of the human body in which there is a forced movement of fluids (body fluids); in the current tests, such phenomena have been omitted.

The PBS extracts of some irradiated biomaterials, such as i.e., PCL_MWCNT_, or PCL_TI_BR_, unexpectedly show marked proliferation stimulatory capacities (Table 1). The observed effect may reflect the increase of nutrients that are available to the assayed NHDF, which is caused by the X-ray induced breakdown of PCL into Krebs-cycle absorbable molecules, or an as yet unidentified phenomenon. The observation requires further investigation.

## 5. Conclusions

The scaffolds containing carbon nanotubes presented here may be used in the future in the design of carriers of anti-cancer substances and become an alternative, or at least a radiotherapy-supporting therapeutic intervention. The potential leaching of materials upon the irradiation of such modified titanium plates, and their effect on normal human dermal fibroblasts (NHDF) have been assessed by MTT assay. The presented results show variable biological responses depending on the modification of titanium plates.

**Figure 8:**
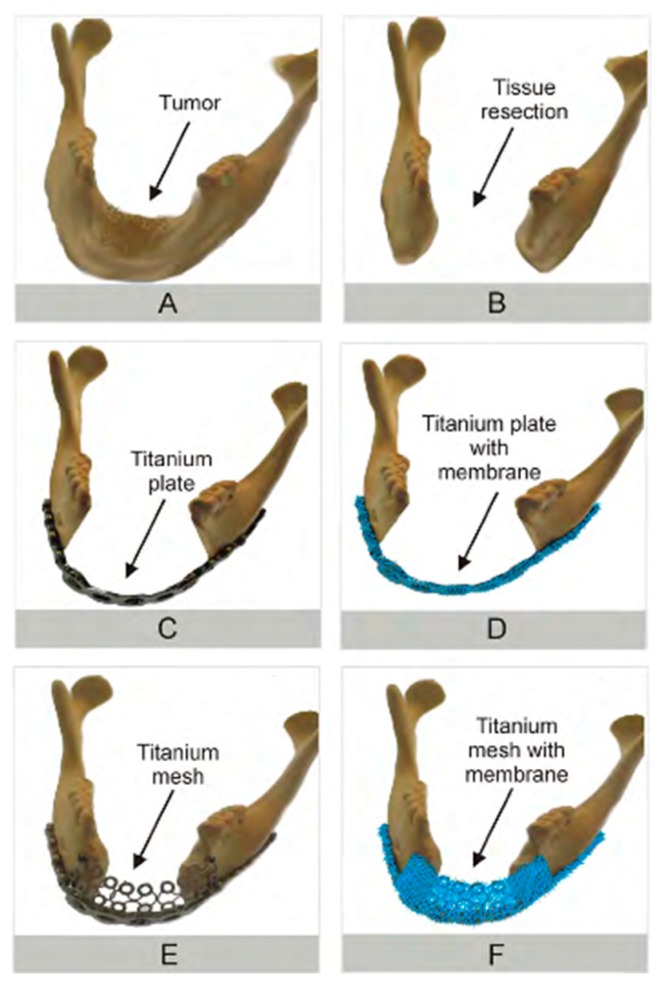
Schematic illustration of various reconstructive procedures within the mandible. (**A**) Mandible before surgery, with tumor indicated by an arrow; (**B**) mandible after surgery, with tumor and adjacent areas removed (please note large safety margin area); (**C**) mandible reconstruction with the use of a unmodified titanium plate; (**D**) suggested mandible reconstruction procedure with the use of a titanium plate covered by a slow-resorbing biomaterial membrane; (**E**) mandible reconstruction with the use of a titanium plate combined with a titanium mesh; (**F**) suggested mandible reconstruction procedure with the use of a titanium plate, and titanium mesh, both covered by slow-resorbing biomaterial membrane.

In order to finally confirm and determine the usefulness of membranes application in the area of maxillofacial surgery in the manner proposed by the authors of this project (Figure 8D,F), in vivo tests are planned. Only then will it be possible to thoroughly evaluate the application possibilities of the obtained scaffolds.

## Figures and Tables

**Table 1:** The survival of NHDF in contact with the tested biomaterials, as assessed by MTT in comparison to the uncoated titanium plates (control).

**I** **Comparison of Cell Survival in Contact with Solutions/Extracts Obtained after Incubation with Membranes**					
**Control Value ± SD**	**Membrane Material**	**MTT Readout Absolute Value; (%)**	**±SD**	**Proliferation Effect**	**NHDF Viability after Incubation with Post-Irradiated 1:1-Diluted PBS, 72 h MTT Assay**
0.0930 (100%) ± 0.0085	PCL_TI_	0.0805 (86.5%)	0.0021	↓ decrease	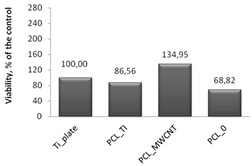
	PCL_MWCNT_	0.1250 (134.4%)	0.0007	↑ increase	
	PCL_0_	0.0640 (68.8%)	0.0000	↓ decrease	
**II** **Comparison of Cell Survival in Contact with Solutions/Extracts Obtained after Incubation with Titanium Plates Covered with Membranes**					
**Control Value Absolute Value; (%) ± SD**	**Membrane Material in Contact with the Titanium Plate**	**MTT Readout Absolute Value; (%)**	**±SD**	**Proliferation Effect**	**NHDF Viability after Incubation with Post-Irradiated 1:1-Diluted PBS, 72 h MTT Assay**
0.0930 (100%) ± 0.0085	PCL_TI_BR_	0.2500; (268%)	0.0226	↑ increase	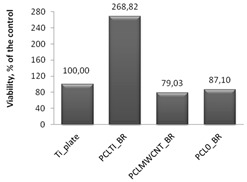
	PCL_MWCNT_BR_	0.0735; (79%)	0.0007	↓ decrease	
	PCL_0_BR_	0.0810; (87%)	0.0042	↓ decrease	
